# Research in Health-Emergency and Disaster Risk Management and Its Potential Implications in the Post COVID-19 World

**DOI:** 10.3390/ijerph18052520

**Published:** 2021-03-04

**Authors:** Emily Ying Yang Chan, Holly Ching Yu Lam

**Affiliations:** 1 JC School of Public Health and Primary Care, Faculty of Medicine, The Chinese University of Hong Kong, Hong Kong, China; 2 Nuffield Department of Medicine, University of Oxford, Oxford OX3 7BN, UK; 3 Genomic and Environmental Medicine, National Heart and Lung Institute, Imperial College London, London SW7 2AZ, UK; ching.lam@imperial.ac.uk

Health-Emergency Disaster Risk Management (Health-EDRM) is one of the latest academic and global policy paradigms that capture knowledge, research and policy shift from response to preparedness and health risk management in non-emergency times [1]. This concept encompasses risk analyses and interventions, such as accessible early warning systems, timely deployment of relief workers, provision of suitable drugs, and medical equipment to decrease the impact of disasters on people before, during, and after an event(s). The approach emphasizes the investment into disaster health risk reduction efforts which may thereby strengthening health systems and capacity to ensure community health resilience building. Health emergency disaster risk management (Health-EDRM) thus refers to the systematic analysis and management of health risks surrounding emergencies and disasters, and plays an important role in reducing hazards and vulnerability along with extending preparedness, response, and recovery measures [1].

Disasters such as earthquakes, cyclones, floods, heat waves, nuclear accidents, and large-scale pollution incidents cost human lives and incur long-term health and well-being implications. The most vulnerable population subgroups in the majority of the disasters often comprise of extreme ages, remote living areas, and endemic poverty, as well as people with low literacy. However, scientific evidence gaps remain in the published literature regarding health risks patterns and cost-effectiveness Health-EDRM risk reduction strategies to facilitate a more efficient reduction of global disaster risks through global policies and initiatives [2,3]. The first Special Issue of *IJERPH*, published in 2018–19 with the thematic focus on Health-EDRM included 20 papers that characterized disaster risks, analysed health risk and interventions effectiveness [4]. The 2nd edition (2019–2020) further compiles 16 scientific papers that have been published in 2020. Papers included in this 2nd edition demonstrate the diverse range of health-related disaster and emergency risk management topics and research analyses that evaluate short- and long-term health impacts, associated risk factors, risk assessment methods and tools as well as multidisciplinary research methods related to program evaluation and policy analysis.

With the complexity and interconnectedness of modern living, multidisciplinary research methodologies development is crucial, as it may enhance the assessment of health risks, impacts and promote understanding of health risks, disaster and humanitarian medicine for non-health stakeholders. The study of Zhang et al. [5] shows how multi-disciplinary methodologies might be applied to examine communicable disease spread among close-contacts. Ma et al. [6] proposes a landslide risk prediction approach that might be useful to prevent mortality and morbidity in at-risk communities.

In the 21st century, the human health impacts of climate change are expected to be significant globally. In this issue, five studies are published to delineate population health risks that may be associated with climate change in various contexts. The studies examine socio-demographic patterns of self-help and community bottom-up health protection strategies during extreme temperature events [7,8] (Lam et al., Liu et al.) and typhoon/hurricane [9,10] (Shang et al., Shih et al.). In addition, with the global increasing health risks associated with vector-borne diseases as a result of climate abnormalities, [11] Chan et al. present a narrative review paper of the current understanding of primary preventive Health-EDRM measures that might reduce the health risks of vector-borne disease in communities.

Another research article subset published in this edition is related to the evaluation of human health and well-being with man-made/technology-related disasters. In their case study, Genereux et al. [12] analyze how mixed-method-based need assessment might capture potential health risks and needs and subsequently enhance effectiveness and relevance response and recovery of the 2013 Lac-Megantic Rail disaster. The study of Orui et al. [13] finds an association between media information and post nuclear accident health anxiety. The findings of this study may inform policymakers and clinical practitioners about mental and health risk reduction strategies. Based on a mixed-method approach, Lorenzoni et al. [14] examines various long-term implications of disasters on public health system performance, security and health protection. 

Psychosocial and mental health risks and impacts are another important research development area of the Health-EDRM. Takahashi et al. and Umeda et al. have examined the acute mental health needs [15] of disaster victims and potentially how to protect and promote the mental risks of responders [16]. Newnham et al. [17] described the activities and strategic plans of a newly established mental health network in Asia Pacific that aims to encourage and build the research agenda of Health-EDRM-related issues in the region.

Among all the major global disasters in 2020, SARS-CoV2, the biological hazard which caused the COVID-19 pandemic, once again showed how health risks may be far-reaching to cause mortality and affect lives in the 21st century. Within a year, the pandemic has accumulated over 110 million cases and 2.5 million deaths [18]. Meanwhile, research and reported experiences in Asia, as the first global region that was hit by the pandemic in early 2020, might be useful to facilitate understanding of health risks and impacts of a new disease of unknown origin. Kim et al. reports on the COVID-19 impact on mental health status of people living in Daegu, South Korea, the community with the 2nd highest rate of COVID-19 beyond Wuhan city PRC China, during the early phase of the pandemic [19]. Chan et al. [20] examines the sociodemographic predictors of health risk perception, attitudes and behavioral practices of the management COVID-19 in a high-density metropolis—HK, China during the first 2 months of the pandemic (6). The same research team also published an article that examines the health risks and situation of people with non-communicable diseases during the pandemic [21].

In the upcoming months, the global research community is expected to receive a significant amount of Health-EDRM research outputs related to the COVID-19 pandemic. With heavy emphasizes on the hierarchy of prevention and adverse disaster risk reduction [22], researchers and policy makers of Health-EDRM should be proactive in the application of the concepts and tools highlighted in the Health-EDRM paradigm and frameworks [23]. Such efforts will improve research effectiveness for strengthening practice and policy making that aim to protect human health and well-being from future epidemics and disasters.
